# ECG-derived spatial QRS-T angle is associated with ICD implantation, mortality and heart failure admissions in patients with LV systolic dysfunction

**DOI:** 10.1371/journal.pone.0171069

**Published:** 2017-03-30

**Authors:** Sarah Gleeson, Yi-Wen Liao, Clementina Dugo, Andrew Cave, Lifeng Zhou, Zina Ayar, Jonathan Christiansen, Tony Scott, Liane Dawson, Andrew Gavin, Todd T. Schlegel, Patrick Gladding

**Affiliations:** 1 Department of Cardiology, North Shore Hospital, Waitemata District Health Board, Auckland, New Zealand; 2 Division of Cardiology, Azienda Ospedaliera Universitaria Integrata, Verona, Italy; 3 Department of Epidemiology and Public Health, North Shore Hospital, Waitemata District Health Board, Auckland, New Zealand; 4 Deparment of Clinical Informatics, North Shore Hospital, Waitemata District Health Board, Auckland, New Zealand; 5 Department of Clinical Physiology, Karolinska Institutet, Stockholm, Sweden; 6 Nicollier-Schlegel Sàrl, Trélex, Switzerland; 7 Theranostics Laboratory, North Shore Hospital, Waitemata District Health Board, Auckland, New Zealand; Kurume University School of Medicine, JAPAN

## Abstract

**Background:**

Increased spatial QRS-T angle has been shown to predict appropriate implantable cardioverter defibrilIator (ICD) therapy in patients with left ventricular systolic dysfunction (LVSD). We performed a retrospective cohort study in patients with left ventricular ejection fraction (LVEF) 31–40% to assess the relationship between the spatial QRS-T angle and other advanced ECG (A-ECG) as well as echocardiographic metadata, with all-cause mortality or ICD implantation for secondary prevention.

**Methods:**

534 patients ≤75 years of age with LVEF 31–40% were identified through an echocardiography reporting database. Digital 12-lead ECGs were retrospectively matched to 295 of these patients, for whom echocardiographic and A-ECG metadata were then generated. Data mining was applied to discover novel ECG and echocardiographic markers of risk. Machine learning was used to develop a model to predict possible outcomes.

**Results:**

49 patients (17%) had events, defined as either mortality (n = 16) or ICD implantation for secondary prevention (n = 33). 72 parameters (58 A-ECG, 14 echocardiographic) were univariately different (p<0.05) in those with vs. without events. After adjustment for multiplicity, 24 A-ECG parameters and 3 echocardiographic parameters remained different (p<2x10^-3^). These included the posterior-to-leftward QRS loop ratio from the derived vectorcardiographic horizontal plane (previously associated with pulmonary artery pressure, p = 2x10^-6^); spatial mean QRS-T angle (134 vs. 112°, p = 1.6x10^-4^); various repolarisation vectors; and a previously described 5-parameter A-ECG score for LVSD (p = 4x10^-6^) that also correlated with echocardiographic global longitudinal strain (R^2^ = - 0.51, P < 0.0001). A spatial QRS-T angle >110° had an adjusted HR of 3.4 (95% CI 1.6 to 7.4) for secondary ICD implantation or all-cause death and adjusted HR of 4.1 (95% CI 1.2 to 13.9) for future heart failure admission. There was a loss of complexity between A-ECG and echocardiographic variables with an increasing degree of disease.

**Conclusion:**

Spatial QRS-T angle >110° was strongly associated with arrhythmic events and all-cause death. Deep analysis of global ECG and echocardiographic metadata revealed underlying relationships, which otherwise would not have been appreciated. Delivered at scale such techniques may prove useful in clinical decision making in the future.

## Background

Patients with left ventricular systolic dysfunction (LVSD) are at increased risk for sudden cardiac death (SCD) due to ventricular arrhythmia. A number of large randomised trials have shown a mortality benefit for the prophylactic use of implantable cardioverter defibrilIators (ICDs) in patients with left ventricular ejection fraction (LVEF) ≤35%, and NYHA Class II, or worse, symptoms. International guidelines therefore recommend the use of primary prevention ICDs in this population[1]. However many middle to low OECD countries have no policy recommendations for the use of primary prevention ICDs, due to limited resources. For instance, in New Zealand patients with LVEF between 30–35% are not considered eligible for an ICD. Furthermore the recent DANISH study has also brought into question the role of primary prevention ICDs in patients with a nonischaemic cardiomyopathy and an LVEF ≤35%[2].

Although LVEF and symptoms are currently considered the main determinants of risk, various advanced ECG (A-ECG) parameters from the resting ECG have also been shown to predict sudden cardiac death and ventricular arrhythmia[3–6]. One such metric, the spatial QRS-T angle, can be calculated from a standard digital 10-sec 12-lead ECG by first deriving the vectorcardiogram from the ECG, and then measuring the 3D angle between the spatial QRS and T-wave maximal or mean loop vectors. This parameter has been shown to have a high value in predicting ventricular arrhythmias not only in patients with left ventricular systolic dysfunction[3, 7], but also in those with normal LVEF[8–11].

While patients with LVEF 35 to 40% are also at risk for ventricular arrhythmia and SCD, they are not eligible for primary prevention ICD therapy under current guidelines, due to cost and an estimated 6% incidence of serious ICD complications[12, 13]. An individualised approach to estimating risk and benefit is therefore necessary in this population. In this retrospective cohort study, we evaluated the utility of the spatial QRS-T angle in predicting outcomes, using a clinical electronic database of patients with LVEF 31 to 40%. We also used agnostic mining of global conventional and A-ECG, as well as echocardiographic metadata, to potentially identify other novel biomarkers of risk for all cause death, secondary prevention ICD implantation and heart failure admissions. This global metadata was also used to evaluate inter-relationships between the data and overall data complexity in patients with and without events.

## Methods

### Patients

All research was approved by the local Institutional Review Board (Waitemata District Health Board Knowledge Centre), and was conducted in accordance with the Declaration of Helsinki (Ethical Guidelines for Observational Studies 2012. Health and Disability Ethics Committees of New Zealand). The IRB waived the requirement for participants to provide consent, as the project was a retrospective clinical audit. All patients who had undergone an echocardiogram between February 2010 and January 2015, with an LVEF of 30.5% to 39.4% via Simpson’s biplane method, were identified using a SQL server search of an Excelera database (Philips Healthcare, Best, The Netherlands). Only those with complete bundle branch block, existing ICD or cardiac resynchronisation therapy (CRT), paced rhythm, or a noisy or absent ECG were excluded.

### Clinical data and outcomes

Clinical information and ICD outcomes were manually identified with the use of electronic medical records. Mortality and heart failure readmissions were identified using International Classification of Diseases 10 (ICD10) queries. A socioeconomic deprivation score was populated using hospital databases. The primary outcomes of interest were prespecified as all cause death and secondary prevention ICD implantation due to a community or hospital cardiac arrest and documented VT/VF. A secondary outcome of interest was heart failure admissions. Spatial mean QRS-T angle was prespecified as the variable of principal interest in relation to all outcomes. Clinical data was matched against A-ECG metadata, post-analysis and re-identification.

### A-ECG analysis

12-lead ECGs, corresponding to the first echocardiogram performed on each patient, were extracted from Epiphany’s Cardio Server ECG management software (Version 3.2.3.1, Epiphany Healthcare, Midlothian, VA, USA), de-identified, and converted to an analysable, binary format by using an Octave script, then sent to Advanced ECG Services (Nicollier-Schlegel SARL, Trélex, Switzerland; https://aecg.ch) for blinded A-ECG analyses. Such analyses were limited to only those parameters that can be accurately assessed from 10-s recordings, and did not include temporal parameters such as heart rate variability and QT variability. Analyzed parameters included those derived from signal averaging of all adequately cross-correlated QRS and T complexes within the 10-s recordings by using software originally assembled at NASA [14, 15] to generate results for: (1) several spatial (derived vectorcardiographic or 3-dimensional) ECG parameters, including the spatial mean and peaks QRS-T angles, the spatial ventricular gradient, and various spatial waveform azimuths, elevations, and time-voltages [15], all derived by using the Frank-lead reconstruction technique of Kors *et al*.[16]; (2) parameters of QRS and T-waveform complexity derived by singular value decomposition (SVD) that can be reproducibly obtained from 10-s ECGs, for example the principal component analysis (PCA) ratio[14] and the dipolar voltage equivalents [15, 17] of the QRS and T waveforms; and (3) the most applicable parameters from the conventional scalar 12-lead ECG. The majority of these parameters and their related detailed methods have been described in previous publications[14–16, 18]. We also calculated results for a previously validated A-ECG score for LVSD that was originally constructed on the basis of multivariate logistic regression of A-ECG data from a larger data set of previous patients with known LVSD[15]. This score specifically incorporates results from a patient's spatial mean QRS-T angle, derived Frank Z-lead QRS integral, total (12-lead) QRS voltage, and SVD-derived QRS-wave nondipolar and T-wave dipolar voltages [15]. The exact coefficients utilized in the score can also be found within Supplemental Table 2 of [15].

### Echocardiography analysis

Echocardiograms were performed as part of clinical care on GE Vivid 7, E9 or Philips IE33 ultrasound machines. All echocardiograms were performed by an experienced sonographer under cardiologist (ASE level III) supervision. Complete echocardiographic metadata, including all sonographer inputted measurements, were extracted using SQL coding and matched to re-dentified A-ECG data post-analysis. NULL values, text fields and low count variables were excluded as part of feature selection. Echocardiographic variables were then compared between patients with versus without the studied primary and secondary outcomes. Left ventricular global longitudinal strain (GLS) analysis was also performed on randomly chosen echocardiograms by using EchoPAC version 113.0.5. These GLS data were enriched by results from a further 24 echocardiograms from patients with normal LVEF to mild LVSD, who were not part of the core study, to encompass a wider spectrum of disease. A subgroup of 102 patients also underwent a second echocardiogram as part of clinical care, and were studied to assess baseline predictors of response to medical therapy. Patients with a >10% increment in LVEF were classified as responders to medical therapy, those with a ≤10% change were classified as nonresponders, and those with >10% decrement were classified as "deteriorators". Primary outcomes for each of these subgroups were also compared. In a posthoc analysis we calculated the aortic energy loss index (ELI) for all patients and compared this between those with and without primary events. The ELI is calculated as (AVA x Aa)/(Aa—AVA), where AVA is derived from the continuity equation and Aa is the aortic area[19, 20].

### Graph analysis and machine learning

After aligning echocardiographic and A-ECG metadata, a feature selected correlation matrix was created from the resulting 291 variables of interest. These data were inputted into Cytoscape 3.3.0, for inter-relational network analysis. An interactive network was generated to compare the metadata of patients with and without a primary outcome. These data were also animated using a Javascript D3 Force layout by generating a correlation matrix from data added one patient at a time, ranked in order by LVEF. The strength of the edges between nodes was proportional to the absolute correlation between related measurements. Correlations for variables with >82 instances were considered statistically significant, based on an adjustment for multiplicity (p<1.2x10^-6^). A statistical certainty index was calculated as the percentage of total variables (n = 291) which reached this threshold. Edges were included for Pearson correlations >0.45 to improve visualisation of the network.

Individual patients were compared by a self-similarity matrix 1:1 to every other patient in the database, using dot product multiscalar vectors and a graph analysis that applied the Javascript D3 Force layout. Machine learning (Decision Tree analysis) was applied to global metadata using BigML (Corvallis, Oregon, USA). For this analysis only, an additional 8 patients who already had secondary ICDs were also added to the database to increase numbers. These patients were identified during the initial SQL query. After randomly splitting these data 60:40 into training and validation sets, multiple prediction models were then compared using ROCs.

### Statistics

A student T test was applied to parametric data to compare patients with versus without primary or secondary outcomes. As patients were identified with an index echocardiogram throughout the 5-year period Kaplan Meier analyses were used for time adjustment to compare outcomes. Receiver Operator Curves (ROCs, Medcalc v 16.4.3) were used to identify the most accurate (combined sensitivity and specificity) cut off values of the spatial mean QRS-T angle for events, with the cut offs then being applied forward to the Kaplan Meier curves. Multiple ROCs were also compared for metadata variables that were significantly different, in those with versus without events. Cox-Proportional Hazard models were used to evaluate models of risk for the primary and secondary outcomes, using SAS version 9.4. For standard statistics a nominal p<0.05 was deemed statistically significant. To correct for multiplicity, a Bonferroni correction was applied with p <1.7x 10^−4^ for 291 variables. Qualitative measures, such as shortest path distribution, network topology, between-ness centrality, degree and network re-wiring (DyNet Cytoscape plugin), were used to evaluate networks.

## Results

### Patient characteristics and outcomes

534 patients ≤75 years of age with LVEF31-40% were identified through the echocardiography reporting database. Digital 12-lead ECGs were matched to 345 of these patients within a timeframe of less than six months. 50 of these patients were excluded due to the presence of one of exclusionary conditions, as noted, leaving 295 for the final analysis. Baseline characteristics are shown in Table 1.

**Table 1:** Baseline characteristics.

	No Event n = 246	Event n = 49	P value
**Age**	60 (12)	64 (9)	0.02
**Male**	181 (70%)	38 (80%)	0.56
**Atrial fibrillation**	74 (30%)	14 (30%)	0.14
**Hypertension**	102 (41%)	25 (51%)	0.2
**Dyslipidaemia**	85 (34%)	21 (43%)	0.29
**Smoker**	62 (25%)	12 (24%)	0.88
**Type 2 Diabetes**	53 (21%)	18 (37%)	0.03
**NYHA**	1.1 (1)	1.3 (0.9)	0.33
**Beta-blocker**	174 (71%)	40 (82%)	0.11
**ICM**	124 (50%)	26 (53%)	0.7
**NICM**	98 (40%)	18 (37%)	0.7
**Valvular, other**	24 (10%)	15 (10%)	1
**LVEF (Simpson)**	36 (2.9)	35 (2.9)	0.04
**Spatial QRS-T angle**	112 (38)	134 (33)	1.6x10^-4^
**Socioeconomic NZDep2006 deprivation score**	5.6 (3)	5.4 (2.6)	0.72

NYHA = New York Heart Association functional classification; ICM = ischaemic cardiomyopathy; NICM = nonischaemic cardiomyopathy; LVEF = left ventricular ejection fraction measured by Simpson biplane; NZDep2006, New Zealand socioeconomic deprivation score, 1 most deprivation, 10 least deprivation. Six patients had aortic and 5 mitral prostheses and 16 had moderate-to-severe aortic stenosis.

144 (49%) of these patients had LVEF 31–35% and151 (51%) had LVEF 36–40%. 49 patients (17%) had primary events, defined as either mortality (n = 16) or ICD implantation for secondary prevention (n = 33) over the 5-year period. 26 patients had admissions for congestive heart failure, with several of those having numerous admissions. Secondary ICDs were implanted for documented sustained ventricular arrhythmic events. The median time from the index echocardiogram until the end of the study was 652 days, range 6 to 1,814 days. There was a significant length time bias as patients with events had echocardiograms earlier in relation to the closure date; 1,099 +/-510 days versus 690 +/-475 days, p = 4x10^-7^.

### Data mining of metadata

72 parameters (58 ECG, 14 echocardiographic) were univariately different (p<0.05) in those with vs. without events. After adjustment for multiplicity, 24 A-ECG parameters and two echocardiographic parameters remained different (i.e., p<1.7x10^-4^). The A-ECG parameters included: The posterior-to-leftward (P/L) QRS loop ratio within the derived vectorcardiographic horizontal plane (2.15 vs.1.15, p = 2x10^-6^, noting that this parameter has previously been correlated to invasively measured pulmonary artery pressures [21, 22]); the spatial mean QRS-T angle (134 vs. 112°, p = 1.6x10^-4^); various repolarisation vectors; and the aforementioned 5-parameter A-ECG score for LVSD (p = 4x10^-6^).[23] The two echocardiographic parameters that after adjustment for multiplicity remained different were the left atrial (LA) volume and the LA/Aortic diameter ratio. Notable amongst the 14 echocardiographic parameters that were univariately different (p<0.05) were the aortic ELI (3.2 versus 2.6, p = 0.005 in those with versus without primary events) and aortic valve area (AVA), the dimensionless severity index (DSI, Vmax)[24], the (LVEDD/BSI), the tricuspid regurgitation (TR) jet maximum velocity, and LVEF.

### Primary and secondary outcomes

**Fig 1:**
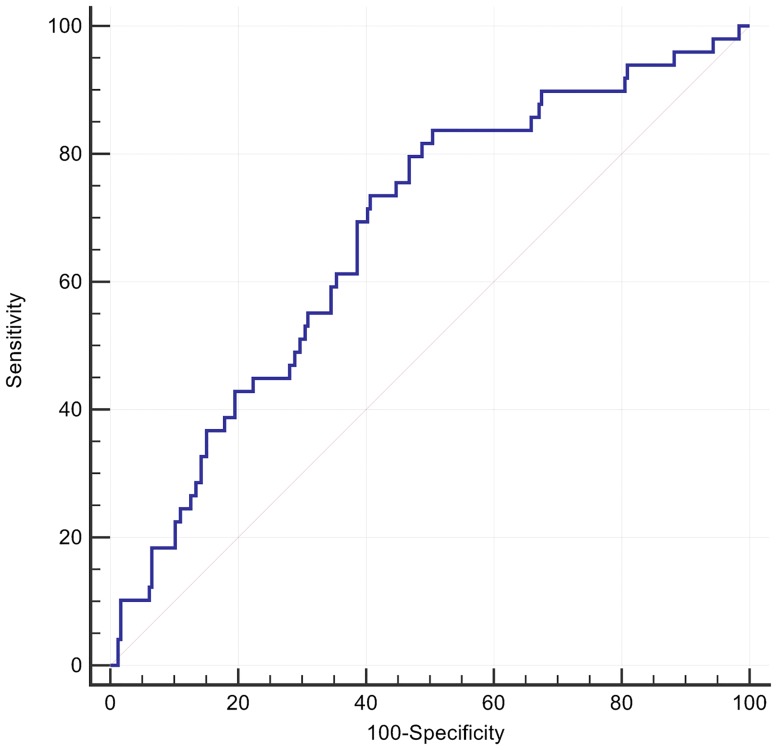
Receiver operator curve for arrhythmic events classified by spatial QRS-T angle. Spatial mean QRS-T angle >110° had a sensitivity 84%, specificity 50% and AUC 0.68 (95% C.I. 0.62 to 0.73, p<0.0001).

**Fig 2:**
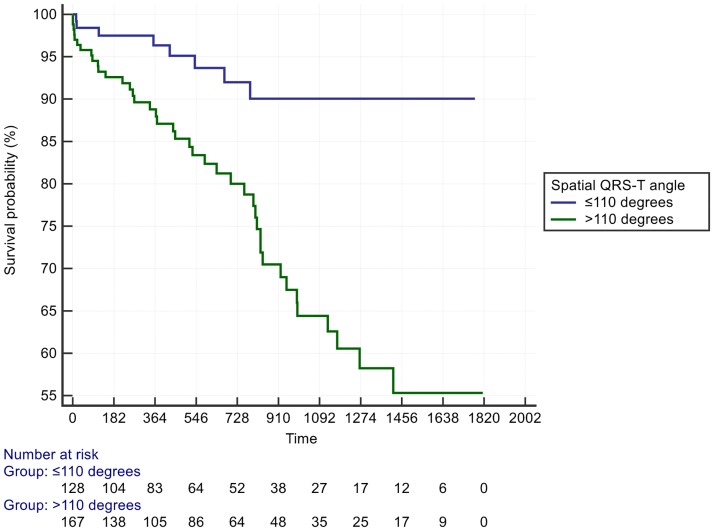
Kaplan Meier plot demonstrating primary event rates over time, dichotomized by a spatial mean QRS-T angle cut off of >110°.

**Fig 3:**
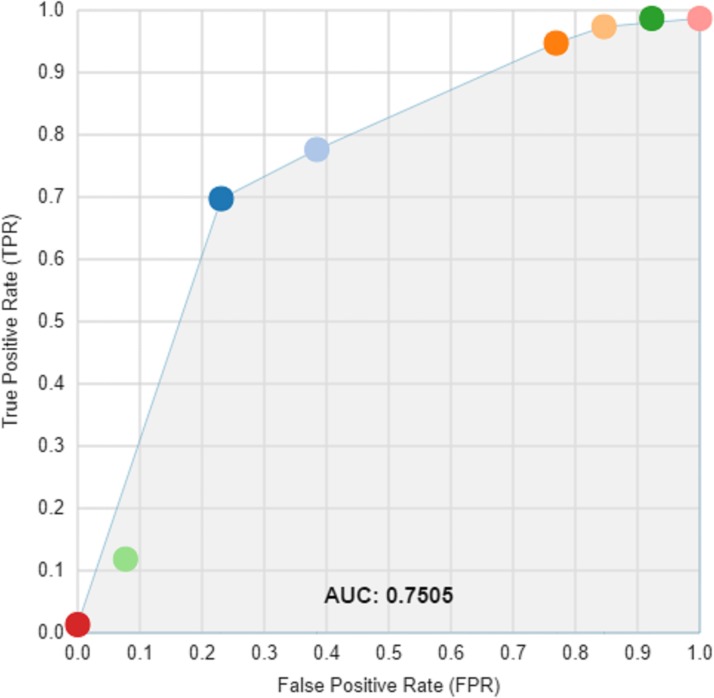
Receiver operator curve for multiple machine learning models, generated using single decision trees and ensembles.

A cut off for the spatial mean QRS-T angle of >110° had the optimal AUC of 0.68 for predicting all-cause death or an arrhythmic event, with accompanying sensitivity of 84% and specificity of 50%, P <0.0001 (Fig 1). Patients with a spatial mean QRS-T angle >110° had a univariate HR = 3.7 (95%CI: 1.7 to 8.0) and adjusted HR of 3.4 (95% CI 1.6 to 7.4, p = 0.001) in a Cox model including type II diabetes and LVEF (Table 2, Fig 2). The effect size was similar in patients with either nonischaemic or ischaemic cardiomyopathy. At its optimal cut off ≤ -2.04, Youden index J 95% CI ≤ -2.46 to ≤ -0.22), the 5-parameter score A-ECG for LVSD, previously validated for its diagnostic superiority over human ECG readers[23], had a higher AUC (0.71) than that of the spatial QRS-T angle for predicting a primary event, although the difference did not reach statistical significance. The LVSD A-ECG score as a continuous variable had an adjusted HR of 0.8 (95% CI 0.7 to 0.98) after adjusting the effects of the binary spatial QRS-T angle result, type II diabetes and LVEF. Machine learning (ML) of global metadata, including 8 patients with prior ICD, further improved the AUC for a primary event to 0.75. By comparison addition of the 8 patients did not alter the AUC for spatial QRS-T angle. Many but not all ML models were dominated by spatial QRS-T angle, and repolarisation parameters. The majority of ML models had high specificity but low sensitivity for primary events (Fig 3).

**Table 2:** Cox Proportional Hazard model for primary event.

	Hazard ratio	95% Confidence Interval
Spatial mean QRS-T angle >110 degrees	3.4	1.6 to 7.4
Type II Diabetes	1.6	0.9 to 2.9
LVEF	0.99	0.9 to 1.1

**Fig 4:**
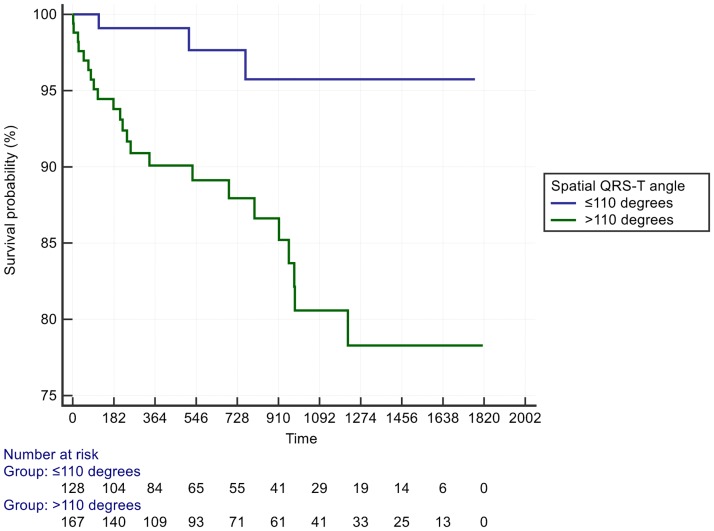
Kaplan Meier plot demonstrating heart failure admissions over time separated by spatial mean QRS-T angle >110°.

**Fig 5:**
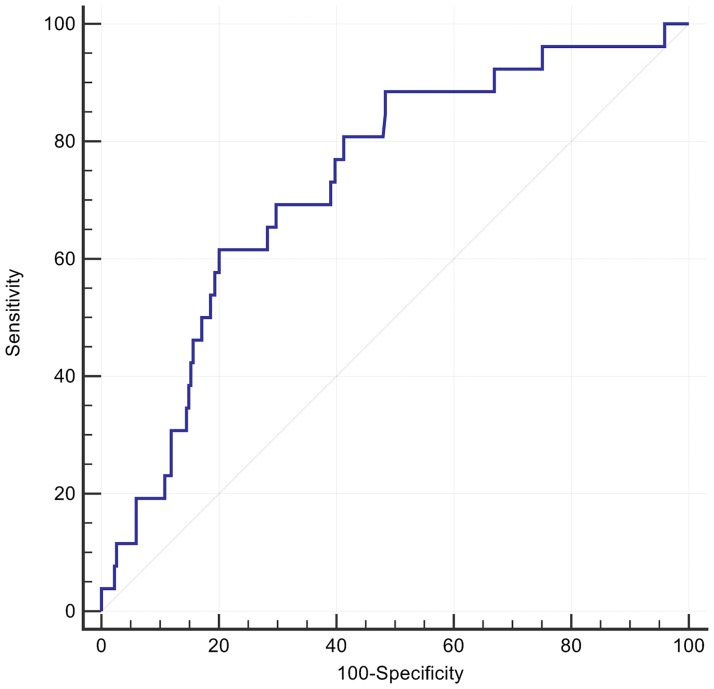
Receiver operator curve for heart failure admissions classified by spatial mean QRS-T angle. Spatial mean QRS-T angle >147° had a sensitivity 64%, specificity 80% and AUC 0.74 (95% C.I 0.68 to 0.79, p<0.0001).

**Fig 6:**
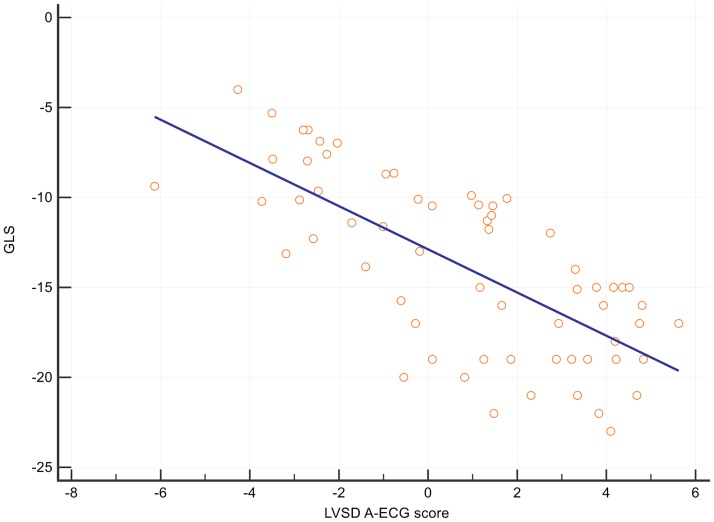
Scatter plot and regression line demonstrating relationship between global longitudinal strain and A-ECG 5-parameter LVSD score.

Patients with a spatial mean QRS-T angle >110° had a HR = 4.1 for the secondary outcome of heart failure admission (95% CI 1.2 to 13.9) (Table 3, Fig 4). The optimal cut off for spatial mean QRS-T angle to predict heart failure admission was >147° (Fig 5). The spatial mean QRS-T angle was also associated with the number of heart failure admissions in a dose-response type of relationship (S1 Fig). Finally, there was also a significant correlation between the A-ECG LVSD score and echocardiographic global longitudinal LV strain, R^2^ = - 0.51, P < 0.0001 (Fig 6).

**Table 3:** Cox Proportional Hazard model for secondary event.

	Hazard ratio	95% Confidence Interval
Spatial mean QRS-T angle >110 degrees	4.1	1.2 to 13.9
Type II Diabetes	2.3	1.0 to 5.1
Age	1.1	1.0 to 1.1

### Spatial mean QRS-T angle >110°

A spatial mean QRS-T angle >110° was associated with numerous structural and functional abnormalities, such as a larger left ventricular end diastolic dimension (LVEDD) (60mm vs 57mm, p = 5x10^-5^) and left ventricular end systolic dimension (LVESD), higher LV mass, lower LVEF (35% versus 36%), stroke volume index and DSI (Vmax). LA diameter was larger and there was a higher prevalence of type II diabetes and hypertension in those with spatial QRS-T angle >110°.

### Response to medical therapy

Primary outcome events occurred in 0% of responders (n = 26, increase in LVEF >10%), 17% of nonresponders (n = 63, ΔLVEF ≤10%), and 31% of deteriorators (n = 13 decrease in LVEF >10%). After excluding one patient with aortic stenosis, the parameter that best predicted response to medical therapy was the echocardiographic DSI Vmax, which was lower in either nonresponders or deteriorators compared to responders (0.61 versus 0.75, p = 0.0003). A DSI >0.64 predicted likelihood of response with a sensitivity of 90%, specificity of 58% and AUC of 0.75 (95% C.I. 0.6 to 0.8, p<0.0001).

Patients with non-ischaemic cardiomyopathy were also more likely to respond than those with ischaemic cardiomyopathy. Responders had higher heart rates, a lower prevalence of type II diabetes, and shorter LV length, than nonresponders or deteriorators.

### Graph analysis

**Fig 7:**
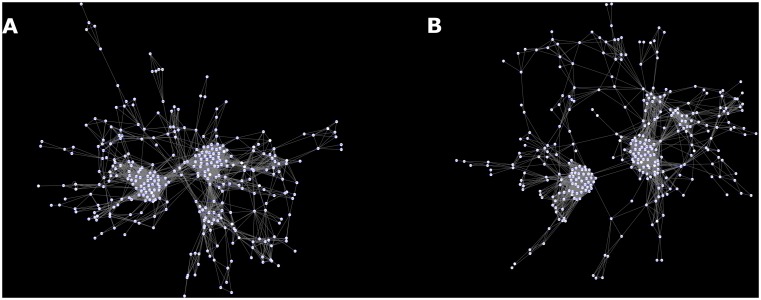
(a) Metadata network of patients without arrhythmic events, (b) all patients including those with events, demonstrating reduced complexity and increased path length.

Parameters (nodes) that were significantly different in those with primary events were often hubs, with high between-ness centrality. These included the spatial QRS-T angle, the sum absolute QRST integral (SAI QRST) [6, 25], the posterior-to-lateral (P/L) QRS loop ratio[21], and the 5-parameter A-ECG score for LVSD[23]. All of these parameters had high rewiring scores when comparing patients with primary events to those without events. Numerous inter-relationships (edges) between A-ECG and echocardiographic parameters were demonstrated using a network model. The greatest densities of these edges were between LV structural parameters, such as the LVEDD/BSI, LV mass and measures of cardiac output, such as the cardiac index. Atrial ECG parameters such as P duration, P wave axis in the frontal plane, and PQ interval were key nodes, connecting both echocardiographic and ECG parameters. These relationships weakened, with dissociation of ECG and echocardiography parameters as the LVEF worsened and when adding patients with a primary event, showing a loss of data complexity (Fig 7) (S1 Source code and S1 HTML https://theranosticslab.github.io/NetworkVisualization/) (S1 GIF). This alteration in network topology resulted in an increase in the shortest path length and its distribution.

**Fig 8:**
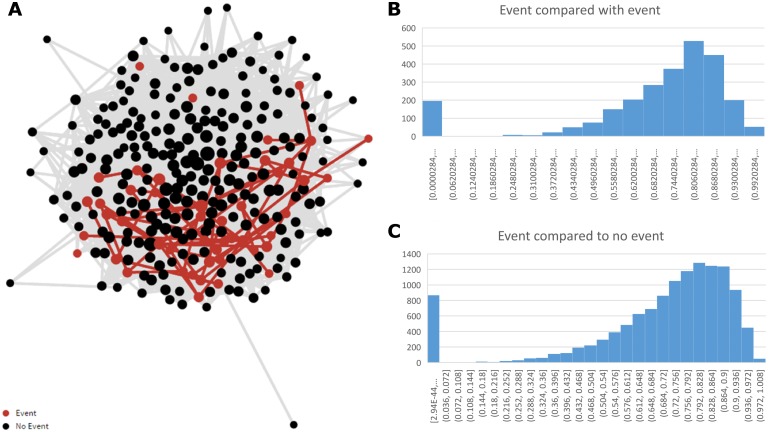
Self-similarity network (a), and distributions (b & c) showing similarity between patients with primary events and between those with versus without primary events.

The dot product similarity matrix demonstrated that patients were very similar to each other, and these relationships followed a skewed, power law distribution. Patients with events were more similar to other patients with events than to those without events, suggesting a phenotypic distinction within the former group; median similarity 0.79 versus 0.76, P<0.0001. Patients without events were more heterogeneous, and less similar than those with events. (Fig 8).

## Discussion

In this cohort study of patients ≤75 years of age with LVEF 31 to 40%, we have further validated the utility of the spatial QRS-T angle as a prognostic indicator, in our case for the combined outcome of all-cause mortality or secondary ICD implantation. Using agnostic datamining and correction for multiple hypothesis testing, we have also identified novel A-ECG markers of arrhythmic risk, such as the derived vectorcardiographic posterior-to-leftward (P/L) QRS loop ratio in the horizontal plane, previously associated with pulmonary artery pressure measured by right heart catheterisation[21, 22]. This particular finding was also corroborated by our echocardiographic metadata, with the maximal TR jet velocity also being significantly associated with primary events, albeit only univariately. Overall these findings are consistent with previous reports of pulmonary artery hypertension and associated RV dysfunction being independently and additively associated with poorer prognosis in patients with heart failure[26, 27].

While ECG parameters dominated in the prediction of arrhythmic outcomes, numerous echocardiographic parameters were also univariately different in those with versus without events and further improved ML prediction models, when incorporated. The most notable of these echocardiographic parameters was the DSI, which was also a strong predictor of response to medical therapy, even after accounting for patients with aortic stenosis. The DSI values in most patients were high, i.e.,>0.5, discounting what would normally be considered a significant aortic valve gradient. We speculate that this may represent either an increasing demand on the failing heart from fluid energy loss due to turbulence, as blood transits the aortic valve, or an epiphenomenon. The aortic valve energy loss index (ELI) in a posthoc analysis was also univariately higher in patients with versus without primary events. It seems possible that in patients with heart failure, energy loss due to turbulence may be a sign of poorer cardiac function, or it might indicate increased cardiac energy demand on the failing heart, even for low gradients[28]. Alternatively, aortic valve sclerosis may indicate a poorer prognosis due to more advanced coronary artery disease[29]. Computational modelling of heart mechanics and flow dynamics might assist with further validation of this concept[30].

The spatial QRS-T angle has been shown to be associated with SCD, ventricular arrhythmia and heart failure outcomes in a wide range of cardiac conditions, including not only heart failure but also acute coronary syndrome, hypertrophic cardiomyopathy and stable coronary artery disease[31–33]. Furthermore, the spatial QRS-T angle has been shown to be increased in patients with various cardiac risk factors, including smoking, diabetes and hypertension[34–37]. The latter two associations were also observed in this study. It has been proposed that increases in spatial QRS-T angle may be a sensitive indicator of left ventricular electrical remodelling, which can manifest before overt conventional ECG or echocardiographic signs of left ventricular hypertrophy become evident[36]. A cardiac MRI study of patients with secondary ICD implants showed that the spatial QRS-T angle was associated with LV function, volume and mass, as well as myocardial scar burden[38]. In the current study we similarly showed relatively increased LV volume and mass and decreased LVEF in those with spatial mean QRS-T angle >110°. In the absence of MRI data on all patients in this study, the spatial QRS-T angle provided additive indirect information on LV scar burden.

Although a wide spatial QRS-T angle may be indicative of structural heart disease it has also been shown to be predictive of SCD, even in those with presumed normal cardiac structure and function[8–11]. In our study the predictive value of the spatial QRS-T angle was observed not only in patients with LVEF 31–35%, but also in those with LVEF 36–40% who would not otherwise be considered eligible for ICDs. The spatial QRS-T angle has been shown to widen not only in pathology such as decreased LVEF, but also with age[11]. In this study a cutoff of >110° had the highest predictive power for future primary events in patients with LVEF 31–40%. However lower cutoffs may also be predictive for events in patients with only mild LV dysfunction, such that we'd propose that LVEF-specific reference ranges also be established for the spatial QRS-T angle(s), to complement such ranges already in existence for age and gender. This proposal is also supported by the results of our network analysis that showed progressive divergence of ECG and echocardiographic parameters, with events occurring predominantly in those who had lost concordance between these values. The notion that disease or event processes are in part due to loss of ‘healthy’ adaption to environmental perturbations and loss of complexity is also supported by molecular studies of cardiovascular disease[39, 40].

Network analysis in this study also demonstrated that both the spatial QRS-T angle and the SAI QRST, another recently proposed marker of arrhythmic risk in patients undergoing ICD implantation for primary prevention[6, 25], were important hubs separated by module clusters. The posterior-to-leftward (P/L) QRS loop ratio from the horizontal plane and the A-ECG score for LVSD also served as such hubs, although these latter two did not deliver significantly independent information from one another and the A-ECG score only marginally further improved the ROC AUC for prediction of primary events. Interestingly the A-ECG score for LVSD was also highly correlated with global longitudinal strain, itself known to be associated with increased risk of ventricular arrhythmia[41]. We have previously demonstrated that the A-ECG LVSD score, which itself incorporates results from the spatial QRS-T angle, has equal diagnostic sensitivity and higher specificity in the diagnosis of heart failure compared to human readers of a 12-lead ECG[23, 42].

Unbiased machine learning using decision tree analysis however did demonstrate an improvement in AUC for prediction of arrhythmic events, when using combined ECG and echocardiographic metadata. These models frequently included the aforementioned A-ECG parameters, although several did not and instead incorporated multiple other A-ECG parameters of repolarisation. This suggests that there may be no single parameter that optimally predicts events, and that a global approach may be more effective in making risk predictions. Such an approach may also be most useful in clinical settings wherein missing data can be a problem[43]. The application of machine learning to broad clinical metadata, looking for intermediate phenotypes or patterns in response to therapy, has significant potential to improve individualised patient care[44]. Similar ‘phenomapping’ has been used in patients with heart failure with preserved ejection fraction (HFpEF) and demonstrated three subclasses of this condition[45]. In one study, frontal planar QRS-T angle dominated in the discrimination of a HFpEF subtype associated with particularly high risk of heart failure hospitalisation or death. The spatial QRS-T angle is widely regarded as more informational than the frontal planar QRS-T angle[46], as well as more powerful for predicting arrhythmic risk[3], though the former requires computational reconstruction of a 3D ECG model, whereas the latter can be easily measured from a conventional 12-lead ECG.

Several other A-ECG parameters such as R-to-R and QT interval variability that are also known to have predictive value for cardiovascular events[4, 47], but that can only be accurately quantified via using longer-duration ECG recordings (e.g., 5-min), could not be included in this study because of our use of only shorter (10-s) recordings. These other parameters, when available, may also provide useful additional information for quantifying risk[4, 48], although additional studies involving longer duration 12-lead ECG would be required to formally evaluate this. Other sources of data such as genomic, metabolomic or other biomarker information may also be additive to a global model of risk. For example, recent studies have demonstrated that metabolomics may not only be diagnostic for heart failure, but also predictive of responses to cardiac resynchronization and ICD therapies[49–52]. Integrating electrobiochemical ‘omic information may yield further predictive information, which cannot be yielded by any individual method separately[53, 54]. As current methods probably demonstrate only the substrate for arrhythmic risk, one logical next step might be to include continuous community-based monitoring of patients via wearable sensors, with inclusion of metabolomic and possibly genomic data to further strengthen risk assessment for events[55–57].

## Conclusion

In this retrospective cohort study, we have demonstrated that the spatial QRS-T angle is a strong predictor of arrhythmic events, all-cause death and heart failure admissions. This was shown to be predictive in a population of patients with both ischaemic and nonischaemic cardiomyopathy. The spatial QRS-T angle was first described in the 1950s[58], however it has never garnered much interest from practising cardiologists. With a recent meta-analysis of 146,000 patients, including data from ICD trials, a significant relationship between a wide spatial QRS-T angle and arrhythmic risk[59], this parameter can no longer be ignored. Both it and A-ECG more generally may play significant roles in individualising the decision for ICD implantation, particularly in cost constrained environments. However, before incorporation into routine practice, further confirmation is required in prospective randomised studies.

## Limitations

As this was a retrospective, clinical cohort study, selection bias may have played a role in influencing the outcomes. The cohort was heterogeneous which makes bias possible, but also aids in translating findings into a real world context. There was a significant length time bias, as some patients were identified earlier in the study period than others. This however is more likely to have underestimated the effect size of a spatial QRS-T angle >110 degrees in patients, who spent limited time in the study period. The outcomes measured in this study depended on adequate electronic clinical records and ICD10 coding. However, as this study was performed in an environment with universal health records, with nationalised databases, in a relatively constrained geography, follow up attainment was high. Machine learning was limited by low numbers with risk of overfitting, however the machine learning part of the study was intended as a demonstration of the types of analyses which could be performed on a greater scale.

## Supporting information

S1 Fig Bar graph of heart failure admissions stratified by spatial QRS-T angle. (DOCX)

S1 Source code Source code for Javascript D3 Force layout of network visualisations. (ZIP)

S1 HTML Interactive network of global metadata. (ZIP)

S1 GIF Network animation of interactions in global metadata. (GIF)

S1 Dataset (XLSX)

## References

[1] Kusumoto FM, Calkins H, Boehmer J, Buxton AE, Chung MK, Gold MR, et al HRS/ACC/AHA Expert Consensus Statement on the Use of Implantable Cardioverter-Defibrillator Therapy in Patients Who Are Not Included or Not Well Represented in Clinical Trials. Circulation. 2014;130(1):94–125. 10.1161/CIR.0000000000000056 24815500

[2] Kober L, Thune JJ, Nielsen JC, Haarbo J, Videbaek L, Korup E, et al Defibrillator Implantation in Patients with Nonischemic Systolic Heart Failure. N Engl J Med. 2016. Epub 2016/08/30. 10.1056/NEJMoa1608029 27571011

[3] Borleffs CJ, Scherptong RW, Man SC, van Welsenes GH, Bax JJ, van Erven L, et al Predicting ventricular arrhythmias in patients with ischemic heart disease: clinical application of the ECG-derived QRS-T angle. Circ Arrhythm Electrophysiol. 2009;2(5):548–54. 10.1161/CIRCEP.109.859108 19843923

[4] La Rovere MT, Pinna GD, Maestri R, Mortara A, Capomolla S, Febo O, et al Short-term heart rate variability strongly predicts sudden cardiac death in chronic heart failure patients. Circulation. 2003;107(4):565–70. Epub 2003/02/05. 12566367 10.1161/01.cir.0000047275.25795.17

[5] Piccirillo G, Magri D, Matera S, Magnanti M, Torrini A, Pasquazzi E, et al QT variability strongly predicts sudden cardiac death in asymptomatic subjects with mild or moderate left ventricular systolic dysfunction: a prospective study. Eur Heart J. 2007;28(11):1344–50. 10.1093/eurheartj/ehl367 17101636

[6] Tereshchenko LG, Cheng A, Fetics BJ, Butcher B, Marine JE, Spragg DD, et al A new electrocardiogram marker to identify patients at low risk for ventricular tachyarrhythmias: sum magnitude of the absolute QRST integral. J Electrocardiol. 2011;44(2):208–16. 10.1016/j.jelectrocard.2010.08.012 21093871 PMC3058724

[7] Pavri BB, Hillis MB, Subacius H, Brumberg GE, Schaechter A, Levine JH, et al Prognostic value and temporal behavior of the planar QRS-T angle in patients with nonischemic cardiomyopathy. Circulation. 2008;117(25):3181–6. 10.1161/CIRCULATIONAHA.107.733451 18574059

[8] Kardys I, Kors JA, van der Meer IM, Hofman A, van der Kuip DAM, Witteman JCM. Spatial QRS-T angle predicts cardiac death in a general population. European Heart Journal. 2003;24(14):1357–64. 12871693 10.1016/s0195-668x(03)00203-3

[9] Aro AL, Huikuri HV, Tikkanen JT, Junttila MJ, Rissanen HA, Reunanen A, et al QRS-T angle as a predictor of sudden cardiac death in a middle-aged general population. Europace. 2012;14(6):872–6. 10.1093/europace/eur393 22183749

[10] Chua KC, Teodorescu C, Reinier K, Uy-Evanado A, Aro AL, Nair SG, et al Wide QRS-T angle on the 12-lead ECG as a Predictor of Sudden Death beyond the LV Ejection Fraction. J Cardiovasc Electrophysiol. 2016. 10.1111/jce.12989 PMC5039018 27094232

[11] Yamazaki T, Froelicher VF, Myers J, Chun S, Wang P. Spatial QRS-T angle predicts cardiac death in a clinical population. Heart Rhythm. 2005;2(1):73–8. 10.1016/j.hrthm.2004.10.040 15851268

[12] Rosenqvist M, Beyer T, Block M, den Dulk K, Minten J, Lindemans F. Adverse events with transvenous implantable cardioverter-defibrillators: a prospective multicenter study. European 7219 Jewel ICD investigators. Circulation. 1998;98(7):663–70. Epub 1998/08/26. 9715859 10.1161/01.cir.98.7.663

[13] Sweeney MO, Wathen MS, Volosin K, Abdalla I, DeGroot PJ, Otterness MF, et al Appropriate and inappropriate ventricular therapies, quality of life, and mortality among primary and secondary prevention implantable cardioverter defibrillator patients: results from the Pacing Fast VT REduces Shock ThErapies (PainFREE Rx II) trial. Circulation. 2005;111(22):2898–905. Epub 2005/06/02. 10.1161/CIRCULATIONAHA.104.526673 15927965

[14] Batdorf BH, Feiveson AH, Schlegel TT. The effect of signal averaging on the reproducibility and reliability of measures of T-wave morphology. Journal of electrocardiology. 2006;39(3):266–70. Epub 2006/03/15. 10.1016/j.jelectrocard.2005.11.004 16529767

[15] Schlegel TT, Kulecz WB, Feiveson AH, Greco EC, DePalma JL, Starc V, et al Accuracy of advanced versus strictly conventional 12-lead ECG for detection and screening of coronary artery disease, left ventricular hypertrophy and left ventricular systolic dysfunction. BMC cardiovascular disorders. 2010;10:28 Epub 2010/06/23. 10.1186/1471-2261-10-28 20565702 PMC2894002

[16] Kors JA, van Herpen G, Sittig AC, van Bemmel JH. Reconstruction of the Frank vectorcardiogram from standard electrocardiographic leads: diagnostic comparison of different methods. European heart journal. 1990;11(12):1083–92. Epub 1990/12/01. 2292255 10.1093/oxfordjournals.eurheartj.a059647

[17] Rautaharju PM, Kooperberg C, Larson JC, LaCroix A. Electrocardiographic predictors of incident congestive heart failure and all-cause mortality in postmenopausal women: the Women's Health Initiative. Circulation. 2006;113(4):481–9. Epub 2006/02/02. 10.1161/CIRCULATIONAHA.105.537415 16449727

[18] Gladding P, Cave A, Zareian M, Smith K, Hussan J, Hunter P, et al Open Access Integrated Therapeutic and Diagnostic Platforms for Personalized Cardiovascular Medicine. Journal of Personalized Medicine. 2013;3(3):203–37. 10.3390/jpm3030203 25562653 PMC4251391

[19] Bahlmann E, Cramariuc D, Gerdts E, Gohlke-Baerwolf C, Nienaber CA, Eriksen E, et al Impact of pressure recovery on echocardiographic assessment of asymptomatic aortic stenosis: a SEAS substudy. JACC Cardiovascular imaging. 2010;3(6):555–62. Epub 2010/06/15. 10.1016/j.jcmg.2009.11.019 20541709

[20] Garcia D, Pibarot P, Dumesnil JG, Sakr F, Durand LG. Assessment of aortic valve stenosis severity: A new index based on the energy loss concept. Circulation. 2000;101(7):765–71. Epub 2000/02/23. 10683350 10.1161/01.cir.101.7.765

[21] Fujita K, Maeda K, Takasugi T, Tsukano Y, Tanaka N. A study on vectorcardiographic criteria for evaluating right ventricular hypertrophy in chronic obstructive pulmonary disease. Jpn Circ J. 1976;40(11):1301–13. 138755 10.1253/jcj.40.1301

[22] Kamphuis VP, Haeck ML, Wagner GS, Maan AC, Maynard C, Delgado V, et al Electrocardiographic detection of right ventricular pressure overload in patients with suspected pulmonary hypertension. J Electrocardiol. 2014;47(2):175–82. 10.1016/j.jelectrocard.2013.10.010 24370072

[23] Johnson K, Neilson S, To A, Amir N, Cave A, Scott T, et al Advanced Electrocardiography Identifies Left Ventricular Systolic Dysfunction in Non-Ischemic Cardiomyopathy and Tracks Serial Change over Time. Journal of Cardiovascular Development and Disease. 2015;2(2):93. 29371514 10.3390/jcdd2020093 PMC5753097

[24] Rahimtoola SH. Determining That Aortic Valve Stenosis Is Severe: Back-to-the-FuturePhysical Examination and Aortic Valve Area Index/Energy Loss Index ≤0.6 cm2/m2. JACC: Cardiovascular Imaging. 2010;3(6):563–6. 10.1016/j.jcmg.2010.02.006 20541710

[25] Tereshchenko LG, McNitt S, Han L, Berger RD, Zareba W. ECG marker of adverse electrical remodeling post-myocardial infarction predicts outcomes in MADIT II study. PLoS One. 2012;7(12):e51812 10.1371/journal.pone.0051812 23251630 PMC3522579

[26] de Groote P, Millaire A, Foucher-Hossein C, Nugue O, Marchandise X, Ducloux G, et al Right ventricular ejection fraction is an independent predictor of survival in patients with moderate heart failure. Journal of the American College of Cardiology. 1998;32(4):948–54. 9768716 10.1016/s0735-1097(98)00337-4

[27] Ghio S, Gavazzi A, Campana C, Inserra C, Klersy C, Sebastiani R, et al Independent and additive prognostic value of right ventricular systolic function and pulmonary artery pressure in patients with chronic heart failure. Journal of the American College of Cardiology. 2001;37(1):183–8. 11153735 10.1016/s0735-1097(00)01102-5

[28] Rodriguez Muñoz D, Markl M, Moya Mur JL, Barker A, Fernández-Golfín C, Lancellotti P, et al Intracardiac flow visualization: current status and future directions. European Heart Journal—Cardiovascular Imaging. 2013. 10.1093/ehjci/jet086 PMC3806582 23907342

[29] Gaibazzi N, Porter TR, Agricola E, Cioffi G, Mazzone C, Lorenzoni V, et al Prognostic value of echocardiographic calcium score in patients with a clinical indication for stress echocardiography. JACC Cardiovascular imaging. 2015;8(4):389–96. 10.1016/j.jcmg.2014.10.014 25797119

[30] Borazjani I, Westerdale J, McMahon EM, Rajaraman PK, Heys JJ, Belohlavek M. Left Ventricular Flow Analysis: Recent Advances in Numerical Methods and Applications in Cardiac Ultrasound. Computational and Mathematical Methods in Medicine. 2013;2013:11. 10.1155/2013/395081 PMC3652115 23690874

[31] Lingman M, Hartford M, Karlsson T, Herlitz J, Rubulis A, Caidahl K, et al Value of the QRS-T area angle in improving the prediction of sudden cardiac death after acute coronary syndromes. Int J Cardiol. 2016;218:1–11. 10.1016/j.ijcard.2016.05.005 27203188

[32] Lipton JA, Nelwan SP, van Domburg RT, Kors JA, Elhendy A, Schinkel AF, et al Abnormal spatial QRS-T angle predicts mortality in patients undergoing dobutamine stress echocardiography for suspected coronary artery disease. Coronary artery disease. 2010;21(1):26–32. Epub 2009/12/10. 10.1097/MCA.0b013e328332ee32 19996961

[33] Cortez D, Sharma N, Cavanaugh J, Tuozo F, Derk G, Lundberg E, et al The spatial QRS-T angle outperforms the Italian and Seattle ECG-based criteria for detection of hypertrophic cardiomyopathy in pediatric patients. J Electrocardiol. 2015;48(5):826–33. Epub 2015/08/16. 10.1016/j.jelectrocard.2015.07.016 26275983

[34] Voulgari C, Moyssakis I, Perrea D, Kyriaki D, Katsilambros N, Tentolouris N. The association between the spatial QRS-T angle with cardiac autonomic neuropathy in subjects with Type 2 diabetes mellitus. Diabet Med. 2010;27(12):1420–9. 10.1111/j.1464-5491.2010.03120.x 21059095

[35] Voulgari C, Tentolouris N, Papadogiannis D, Moyssakis I, Perrea D, Kyriaki D, et al Increased left ventricular arrhythmogenicity in metabolic syndrome and relationship with myocardial performance, risk factors for atherosclerosis, and low-grade inflammation. Metabolism. 2010;59(2):159–65. 10.1016/j.metabol.2009.06.028 19766273

[36] Dilaveris P, Gialafos E, Pantazis A, Synetos A, Triposkiadis F, Gialafos J. The spatial QRS-T angle as a marker of ventricular repolarisation in hypertension. J Hum Hypertens. 2001;15(1):63–70. 11224004 10.1038/sj.jhh.1001129

[37] Dilaveris P, Pantazis A, Gialafos E, Triposkiadis F, Gialafos J. The effects of cigarette smoking on the heterogeneity of ventricular repolarization. American heart journal. 2001;142(5):833–7. Epub 2001/10/31. 10.1067/mhj.2001.118737 11685171

[38] Shi B, Ferrier KA, Sasse A, Harding SA, Larsen PD. Correlation between vectorcardiographic measures and cardiac magnetic resonance imaging of the left ventricle in an implantable cardioverter defibrillator population. Journal of Electrocardiology. 47(1):52–8. 10.1016/j.jelectrocard.2013.06.018 23993862

[39] Drenos F, Grossi E, Buscema M, Humphries SE. Networks in Coronary Heart Disease Genetics As a Step towards Systems Epidemiology. PLoS One. 2015;10(5):e0125876 Epub 2015/05/08. 10.1371/journal.pone.0125876 25951190 PMC4423836

[40] Baffy G, Loscalzo J. Complexity and network dynamics in physiological adaptation: an integrated view. Physiology & behavior. 2014;131:49–56. Epub 2014/04/23. 24751342 10.1016/j.physbeh.2014.04.018

[41] Haugaa KH, Smedsrud MK, Steen T, Kongsgaard E, Loennechen JP, Skjaerpe T, et al Mechanical Dispersion Assessed by Myocardial Strain in Patients After Myocardial Infarction for Risk Prediction of Ventricular Arrhythmia. JACC: Cardiovascular Imaging. 2010;3(3):247–56. 10.1016/j.jcmg.2009.11.012 20223421

[42] Schlegel TT, Kulecz WB, Feiveson AH, Greco EC, DePalma JL, Starc V, et al Accuracy of advanced versus strictly conventional 12-lead ECG for detection and screening of coronary artery disease, left ventricular hypertrophy and left ventricular systolic dysfunction. BMC Cardiovascular Disorders. 2010;10(1):1–11. 20565702 10.1186/1471-2261-10-28 PMC2894002

[43] Miotto R, Li L, Kidd BA, Dudley JT. Deep Patient: An Unsupervised Representation to Predict the Future of Patients from the Electronic Health Records. Sci Rep. 2016;6:26094 10.1038/srep26094 27185194 PMC4869115

[44] Deo RC. Machine Learning in Medicine. Circulation. 2015;132(20):1920–30. 10.1161/CIRCULATIONAHA.115.001593 26572668 PMC5831252

[45] Shah SJ, Katz DH, Selvaraj S, Burke MA, Yancy CW, Gheorghiade M, et al Phenomapping for novel classification of heart failure with preserved ejection fraction. Circulation. 2015;131(3):269–79. 10.1161/CIRCULATIONAHA.114.010637 25398313 PMC4302027

[46] Brown RA, Schlegel TT. Diagnostic utility of the spatial versus individual planar QRS-T angles in cardiac disease detection. J Electrocardiol. 2011;44(4):404–9. Epub 2011/03/01. 10.1016/j.jelectrocard.2011.01.001 21353236

[47] Piccirillo G, Magrì D, Matera S, Magnanti M, Torrini A, Pasquazzi E, et al QT variability strongly predicts sudden cardiac death in asymptomatic subjects with mild or moderate left ventricular systolic dysfunction: a prospective study. European Heart Journal. 2007;28(11):1344–50. 10.1093/eurheartj/ehl367 17101636

[48] Baumert M, Porta A, Vos MA, Malik M, Couderc JP, Laguna P, et al QT interval variability in body surface ECG: measurement, physiological basis, and clinical value: position statement and consensus guidance endorsed by the European Heart Rhythm Association jointly with the ESC Working Group on Cardiac Cellular Electrophysiology. Europace: European pacing, arrhythmias, and cardiac electrophysiology: journal of the working groups on cardiac pacing, arrhythmias, and cardiac cellular electrophysiology of the European Society of Cardiology. 2016;18(6):925–44. Epub 2016/01/30. 10.1093/europace/euv405 PMC4905605 26823389

[49] Wang J, Li Z, Chen J, Zhao H, Luo L, Chen C, et al Metabolomic identification of diagnostic plasma biomarkers in humans with chronic heart failure. Molecular bioSystems. 2013;9(11):2618–26. Epub 2013/08/21. 10.1039/c3mb70227h 23959290

[50] Hunter WG, Kelly JP, McGarrah RW 3rd, Khouri MG, Craig D, Haynes C, et al Metabolomic Profiling Identifies Novel Circulating Biomarkers of Mitochondrial Dysfunction Differentially Elevated in Heart Failure With Preserved Versus Reduced Ejection Fraction: Evidence for Shared Metabolic Impairments in Clinical Heart Failure. Journal of the American Heart Association. 2016;5(8). Epub 2016/07/31. 10.1161/JAHA.115.003190 PMC5015273 27473038

[51] Nemutlu E, Zhang S, Xu YZ, Terzic A, Zhong L, Dzeja PD, et al Cardiac resynchronization therapy induces adaptive metabolic transitions in the metabolomic profile of heart failure. Journal of cardiac failure. 2015;21(6):460–9. Epub 2015/04/26. 10.1016/j.cardfail.2015.04.005 25911126 PMC4456226

[52] Zhang Y, Blasco-Colmenares E, Harms AC, London B, Halder I, Singh M, et al Serum amine-based metabolites and their association with outcomes in primary prevention implantable cardioverter-defibrillator patients. Europace. 2015. Epub 2015/10/27. 10.1093/europace/euv342 PMC5006959 26498162

[53] Shah SH, Newgard CB. Integrated metabolomics and genomics: systems approaches to biomarkers and mechanisms of cardiovascular disease. Circ Cardiovasc Genet. 2015;8(2):410–9. 10.1161/CIRCGENETICS.114.000223 25901039 PMC4408557

[54] Hunter WG, Kelly JP, McGarrah RW 3rd, Kraus WE, Shah SH. Metabolic Dysfunction in Heart Failure: Diagnostic, Prognostic, and Pathophysiologic Insights From Metabolomic Profiling. Curr Heart Fail Rep. 2016;13(3):119–31. 10.1007/s11897-016-0289-5 27216948 PMC5504685

[55] Topol EJ. INDIVIDUALIZED MEDICINE From Pre-Womb to Tomb. Cell. 2014;157(1):241–53. 10.1016/j.cell.2014.02.012 24679539 PMC3995127

[56] Steinhubl SR, Muse ED, Topol EJ. The emerging field of mobile health. Science translational medicine. 2015;7(283):283rv3 Epub 2015/04/17. 10.1126/scitranslmed.aaa3487 25877894 PMC4748838

[57] Walsh JA 3rd, Topol EJ, Steinhubl SR. Novel wireless devices for cardiac monitoring. Circulation. 2014;130(7):573–81. Epub 2014/08/13. 10.1161/CIRCULATIONAHA.114.009024 25114186 PMC4135373

[58] Urschel DL, Abbey DC. Mean spatial vectorcardiography; the normal QRS and T vectors. American heart journal. 1953;45(1):65–76. Epub 1953/01/01. 13007657 10.1016/0002-8703(53)90007-6

[59] Zhang X, Zhu Q, Zhu L, Jiang H, Xie J, Huang W, et al Spatial/Frontal QRS-T Angle Predicts All-Cause Mortality and Cardiac Mortality: A Meta-Analysis. PLoS One. 2015;10(8):e0136174 10.1371/journal.pone.0136174 26284799 PMC4540436

