# Supplementary material for: ECG-derived spatial QRS-T angle is associated with ICD implantation, mortality and heart failure admissions in patients with LV systolic dysfunction
Source: PLoS One. 2017 Mar 30;12(3):e0171069. doi: 10.1371/journal.pone.0171069 (PMC5373522; doi:10.1371/journal.pone.0171069)
Supplement: S1 Fig — (DOCX) [file pone.0171069.s001.docx]

**SUPPLEMENTAL MATERIAL**
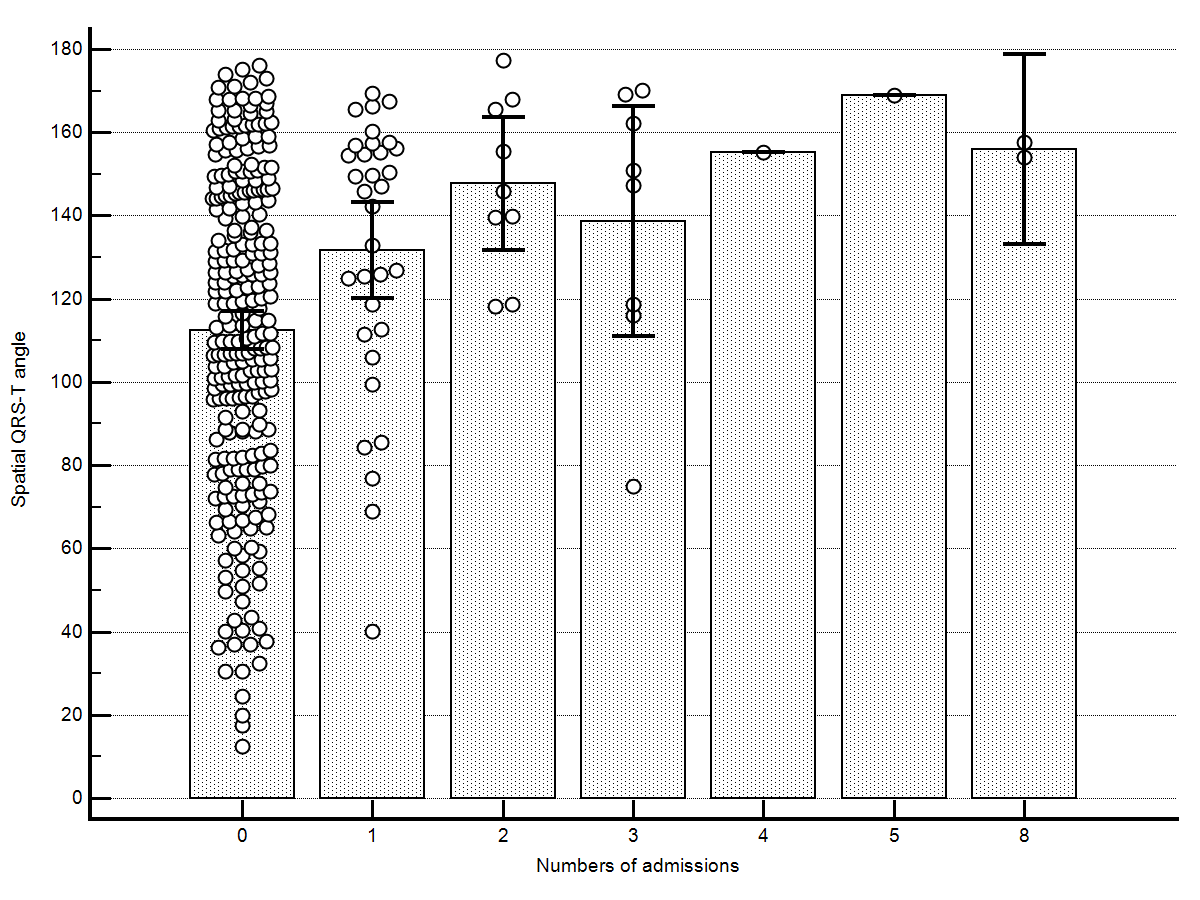


Figure 1. Spatial QRS-T angle and number of number of heart failure admissions per patient.

Movie 1. This GIF shows the inter-relationships between the echocardiographic and ECG metadata, in a series of patients ranked by their EF. As the number of patients increases, and the correlations in the data become statistically significant, a network structure is visible. The complexity and topology of the network changes as more patients, with reducing EF, are added. Finally after adding patients with an arrhythmic event there is a dramatic loss of complexity.
