# Supplementary material for: ECG-derived spatial QRS-T angle is associated with ICD implantation, mortality and heart failure admissions in patients with LV systolic dysfunction
Source: PLoS One. 2017 Mar 30;12(3):e0171069. doi: 10.1371/journal.pone.0171069 (PMC5373522; doi:10.1371/journal.pone.0171069)
Supplement: S1 Source code — (ZIP) [file pone.0171069.s002.zip › Source Code Package/2. Interactive All Data - No Event /Network.html]

[0.45:1.00]

Find Node:


Highlight:
